# Relationship between Psychosocial Risk Factors and Work-Related Musculoskeletal Disorders among Public Hospital Nurses in Malaysia

**DOI:** 10.1186/s40557-014-0023-2

**Published:** 2014-08-09

**Authors:** Nur Azma Amin, Rusli Nordin, Quek Kia Fatt, Rahim M Noah, Jennifer Oxley

**Affiliations:** 1 Jeffrey Cheah School of Medicine and Health Sciences, Monash University Malaysia Jalan Lagoon Selatan, Bandar Sunway, Selangor Darul Ehsan, 46150, Malaysia; 2 Universiti Kuala Lumpur, Institute of Medical Science Technology Jalan TKS 1, Taman Kajang Sentral, Kajang, 43600, Selangor, Malaysia; 3 Monash Injury Research Institute Building 70, Monash University, Victoria 3800, Australia

**Keywords:** Work-related musculoskeletal disorders (WRMSDs), Psychosocial risk factors, Job strain model, Public hospital nurses

## Abstract

**Objective:**

This study examined the relationships between psychosocial work factors and risk of WRMSDs among public hospital nurses in the Klang Valley, Malaysia.

**Methods:**

We conducted a cross-sectional study among 660 public hospital nurses. A self-administered questionnaire was used to collect data on the occurrence of WRMSDs according to body regions, socio-demographic profiles, occupational information and psychosocial risk factors. 468 questionnaires were returned (response rate of 71%), and 376 questionnaires qualified for subsequent analysis. Univariate analyses were applied to test for mean and categorical differences across the WRMSDs; multiple logistic regression was applied to predict WRMSDs based on the Job Strain Model’s psychosocial risk factors.

**Results:**

Over two thirds of the sample of nurses experienced discomfort or pain in at least one site of the musculoskeletal system within the last year. The neck was the most prevalent site (48.94%), followed by the feet (47.20%), the upper back (40.69%) and the lower back (35.28%). More than 50% of the nurses complained of having discomfort in region one (neck, shoulders and upperback) and region four (hips, knees, ankles, and feet). The results also revealed that psychological job demands, job strain and iso-strain ratio demonstrated statistically significant mean differences (p < 0.05) between nurses with and without WRMSDs. According to univariate logistic regression, all psychosocial risk factors illustrated significant association with the occurrence of WRMSDs in various regions of the body (OR: 1.52–2.14). Multiple logistic regression showed all psychosocial risk factors were significantly associated with WRMSDs across body regions (OR: 1.03–1.19) except for region 1 (neck, shoulders and upper back) and region 4 (hips, knees, ankles, and feet). All demographic variables except for years of employment were statistically and significantly associated with WRMSDs (p < 0.05).

**Conclusions:**

The findings indicated the high prevalence of WRMSDs in many body regions, and the risks of developing WRMSDs according to the various body regions were associated with important psychosocial risk factors based on the job strain model. These findings have implications for the management of WRMSDs among public hospital nurses in the Klang Valley, Malaysia.

## Introduction

Injuries and illnesses sustained in the workplace are a major global source of ill health and disability. Worldwide, an estimated two million men and women die each year as a result of work-related injuries or illnesses and a further 268 million non-fatal workplace injuries result in time off work each year [1]. It is also estimated that there are 160 million new cases of work-related illnesses each year. International figures demonstrate the burden of workplace injuries and illnesses. For example, the Bureau of Labour Statistics [2], reported that there were 112 cases per 10,000 fulltime workers requiring days away from work due to non-fatal occupational injuries or illnesses. This includes 34 percent of employees who sustained work related musculoskeletal disorders (WRMSDs) [2]. In Malaysia, more than 50,000 injuries occurred in the workplace every year. In 2012, from more than twelve million workers, there was an estimated one thousand deaths [3], 1,792 reported occupational diseases, but only 95 reported cases related to WRMSDs [4].

WRMSDs are best described as disorders or discomforts experienced by the worker on the musculoskeletal, peripheral nervous, and neurovascular systems, due to prolonged workplace hazards exposure. People who suffer from these types of injuries often experience severe muscle pain that makes simple movements difficult and painful, and the most common type of musculoskeletal injury is the back and neck. The Bureau of Labour Statistics [2] ranked nursing among the occupations with the highest frequency of suffering from WRMSDs, with reported annual prevalence at any of body region varying between 40%-85% [3],[4] among both Asian populations [5]–[8] and Western populations [9]–[11]. Further, the evidence suggests that the most common body regions injured are the lower back area (prevalence rates of 29%-64%) [5],[12], neck (prevalence rates of 34%-54%) [5],[6],[11], and shoulder (prevalence rates of 35%-60%) [8],[10]. In addition, female staff were more susceptible to WRMSDs compared with male staff [7].

The costs associated with a work-related injury or illness is significant: they are not only borne by the individual worker, but also the employer, and society [13]. Costing estimates are generally based on direct costs and include medical expenses (hospitalization, doctors’ visits and rehabilitation), legal costs, and the cost of hiring a replacement worker. Indirect costs, accounting for approximately 75 percent of overall costing, include lost output due to reduced productivity, reduced staff morale, the administration of workers’ compensation claims, and, for the individual, social, economic and psychological difficulties; however, these costs are rarely considered [13]. It is estimated that four percent of the world’s gross domestic product is lost with the cost of injury, death and disease through absence from work, sickness treatment, disability and survivor benefit [1]. In Malaysia alone, one work-related death is estimated to cost RM1.2million in compensation, while a work-related injury resulting in one permanent disability costs RM120,000. In 2010, the total disbursement of temporary disablement was RM109 million, for permanent disability benefits, it amounted to RM306 million, and dependent benefits cost RM205 million [3]. Trinkoff et al. [10] found that 6%, 8%, and 11% of American registered nurses have changed jobs due to neck, shoulder, or back pain, respectively.

A high proportion of workplace incidents within the nursing profession involves WRMSDs which are often a result of laborious tasks including poor manual handling and lifting techniques (such as transferring patients from and to bed, poor body postures), and repetitive and monotonous movements [5],[10]. Psychosocial risk factors [including psychological job demand (PJD), job control/decision latitude (DL) and social support (SS)] were used to denote occupational and organizational factors that were non-physical. PJD refers to the pressure perceived in delivering tasks within short timeframes, while DL is defined as the sum of decision authority and skill discretion. Decision authority refers to the worker’s decision and autonomy in the workplace whereas skill discretion refers to the diversity in assigned tasks [14]. Further, SS denotes support provided in the workplace by peers and supervisors [15]. Previous literatures suggested that interactions of psychosocial factors and physical exhaustion [14] have potentially increased the risk of musculoskeletal pain among nurses [5],[7],[8],[16]. In accordance, a systematic review also found a positive supporting evidences on the association between psychosocial risk factors of limited job control and insufficient peers supports and musculoskeletal disorders [17]. Larsman & Hanse [18] observed the combination of effects of psychosocial risk factors (DL, PJD and SS) in anticipating WRMSDs among female service workers. The high strain group of workers (high PJD, low DL, and low perceived SS) were at greater risk of back, shoulders and neck discomfort (1.80 to 2.06 times) as compared to those working in a more favourable strain environment. Moreover, those categorized as being part of a passive work group (low PJD and DL) but also receiving high SS were at highest possible risk for neck (OR: 2.36, 95% CI: 1.20-4.63) and shoulder (OR: 2.19, 95% CI: 1.05-4.54) pain, in consonant with Karasek’s job demand and control model (JDC model) [14],[19]. It is possible that these workers may have also suffered from job dissatisfaction [19],[20]. Although number of literatures showed the association between WRMSDs and psychosocial factors, yet the evidence relationship is still greatly debated [9],[16]–[18],[21],[22].

Despite a significant body of research documenting the association between psychosocial risk factors and WRMSDs among nurses in developed countries, there is limited research with regards to the nursing populations in Malaysia. Hence, the present study was undertaken to explore the 12-month prevalence of WRMSDs and to investigate the relationship between psychosocial risk factors in the workplace and the risk of sustaining WRMSDs among full time nurses working in public hospitals in the Klang Valley, Malaysia.

## Materials and methods

### Study design and recruitment process

A cross-sectional study design was employed using a sample of female nurses only to avoid gender confounding due to the low number of male nurses. Female nurses aged 23–50 years old, working in shifts, and with a minimum of one year experiences working in the clinical area were invited to participate in the study. Exclusion criteria included: i) nurses with history of related musculoskeletal disorders prior to the study; and ii) nurses who were pregnant or at menopausal stage during data collection. The study took place in four main public hospitals in the Klang Valley. The selection of the hospitals was based on convenience sampling and support received from the respective hospital management. Initial permission to carry out the study was granted by the Director of the respective hospitals. Participants were recruited with the assistance of the Chief Matron at each participating hospital. The study received ethics approval from the Monash University Human Research Ethics Committee (MUHREC), and the Ministry of Health, Malaysia Research Ethics Committee (MREC).

Upon receiving approval from the relevant authorities, and in collaboration with the Chief Matron’s office at the respective hospitals, a briefing session was conducted to potential nurses. During this session, the subject information documents and informed consent forms were distributed to the nurses; interested participants were to submit the informed consent forms by the end of the briefing session. The study package was later distributed to those who consented to participate in the study, through the Nurse Manager. The participants remained anonymous and were identified with special identification codes which were made known only to the research team. The completed self-administrated questionnaires (SAQs) were returned within a week in sealed envelopes and deposited into a locked box located at the Chief Matron’s office. The research team then checked the completeness of the submitted questionnaires and tokens of appreciation were given to each participant.

### Sample size

Sample size was calculated using the single proportion formula with 95 percent confidence interval [23]. Based on the 79 percent prevalence of WRMSDs [24] and low back pain [25] among nurses in a previous study with precision of 4 percent, the estimated sample size was 264. After considering an 80 percent response rate, the study aimed to recruit a minimum sample of 330.A total of 468 completed questionnaires were received from 660 sets of questionnaires, representing a response rate of 70.9 percent. A further 92 questionnaires were excluded due to non-fulfilment of the inclusion criteria. Therefore, a total of 376 sets of questionnaires were available for analysis.

### Research materials

Data was collected using a set of validated and Malay-translated SAQs, consisting of three sections, as follows:

### Demographic and work-related characteristics

This section consisted of information on socio-demography (age, marital status, educational level) and job information (year of employment, hours of work per week).The body mass index (BMI kg/m^2^) was calculated based on self-reported values of weight and height.

### Psychosocial risk factors

A Malay-translated Job Content Questionnaire (M-JCQ) [15],[26] was used to gather information of various psychosocial aspects of the job. A total of 25items from the JCQ’s full recommended format [15] were selected that consisted of four (4) subscales: job control/decision latitude (DL) (9 items); social support (SS) (8 items); psychological job demand (PJD) (5 items); and, job insecurity (JI) (3 items). The items were scored using a Likert scale, ranging from1 (strongly disagree) to4 (strongly agree), and were calculated using Karasek’s recommended formulae [15]. In addition, the scores for the following four items were reversed; one item for decision authority (Q8: “little decision freedom”), and three items of psychological demands (Q22: “no excessive work”, Q23: “enough time”, and Q26: “conflicting demands”). The range of scores were as follows: DL (24–96), PJD (12–48), JI (3–14), and SS (8–32) with higher subscale scores indicating increased severity of the subscales [15].

Next, to assess psychological stress, the median values of the sample for DL, PJD, JI and SS were used to dichotomize the scale into two categories (high/low) [15]. Further, the dichotomized values (high/low) for DL and PJD were later divided into the four job strain quadrants: high strain (high PJD × low DL), low strain (low PJD × high DL), active job (high PJD × high DL) and passive job (low PJD × low DL) [14]. Also, the continuous score is used to calculate the quartiles of DL and PJD and categorized into three groups (low, medium, high) to estimate the job strain [27]. In addition, the iso strain observed the combination effects of job strain and SS [19],[27].

### Assessment of symptoms of WRMSDs

A Malay-translated Standardized Nordic Questionnaire (SNQ-M), based on the original version developed by Kuorinka et al. [28] was used to assess the symptoms of WRMSDs. An anatomical diagram of nine body regions (neck, shoulders, upper and lower back, hands/wrists, arms, knees, thighs, and feet) were appended to facilitate precise identification of the occurrences of discomfort or pain in the previous 12-months period as reported by the respondents [28]. Next, the nine body regions of the musculoskeletal system were grouped into four regions to facilitate analysis: region one (neck, shoulders, and upperback), region two (arms and wrists), region three (lower back), and region four (hips, knees, ankles, and feet) [7]. In addition, the participants were asked to describe the pain level, following the symptoms for the past one year, based on a 5-point pain scale from “0-none/no pain” to “4-worst pain ever” [10],[28],[29]. Nurses presenting with any symptom (pain, numbness, tingling, aching, stiffness, or burning) and scored pain intensity of at least three on a 5 point scale (moderate) in at least one body area in the past one year that persisted at least one week or occurred monthly, were identified as having WRMSDs [28],[29].

### Statistical analysis

Data entry and analysis were undertaken using the IBM SPSS Statistics version 22.0. The data were checked for completeness and examined for normality distribution using the stem-and-leaf plot and the Kolmogorov-Smirnov Test. For continuous parameters, means and standard deviations were computed for normally distributed variables while frequencies and percentages were computed for ordinal and nominal data. The occurrence of WRMSDs was presented as prevalence rate. Next, the association between the dependent variable (WRMSDs) and independent variables (demographic, occupational, and psychosocial risk factors) were assessed using both univariate and bivariate analyses. The chi-square test was used to observe the association between the outcome measures with categorical variables, while the independent t-test was applied to assess the association between the outcome measures with continuous data. The statistically significant demographic variables in the univariate analysis were defined as confounding variables and adopted as covariates. Finally, multiple logistic regression was performed to identify the psychosocial risk factors of WRMSDs, using odds ratios (ORs), 95% CIs, and probability (P) values (set at p < 0.05). All variables in the regression model were also analysed simultaneously to observe the interactions between the psychosocial risk factors.

## Results

### Demographic profiles

Details of the socio-demographic and occupational information are presented in Table 1. The overall mean (SD) age of the participants was 30.61 (5.29) years and the majority were Malays (94.21%) and married (76.41%). The mean (SD) years of employment as a nurse was 7.31 (5.16) years with average working hours/week (SD) of 45.03 (5.43) hours/week. The mean (SD) BMI was 24.19 (4.48).Initial analysis observed a non-significant difference of demographic variables between nurses with and without WRMSDs except for marital status (p = 0.09). Nonetheless, further analysis has statistically signified association between a number of demographic variables with the presence of pain across body regions (p < 0.05).

**Table 1:** Demographic and occupational profile of respondents

**Variables**	**WRMSDs (n = 376)**		**P-value**^**+**^
	**Yes (n = 275)**	**No (n = 101)**	
	Mean (SD)		
Age (yr)	29.62 (7.13)	30.21 (6.70)	0.46
Years of employment as nurses	7.33 (4.89)	7.61 (5.18)	0.64
Working hours/week	45.17 (5.17)	44.65 (5.17)	0.41
Body mass index (BMI) (kg/m^2^)	24.39 (4.49)	23.65 (4.44)	0.15
	% (n)		
Race			
Malay	94.20 (260)	94.10 (95)	0.96
Non-Malay	5.80 (15)	5.90 (6)	
Marital status			
Married	78.91 (217)	70.30 (71)	0.09*
Not married	21.09 (58)	29.70 (30)	

*indicate statistically significance differences.

^+^calculated by t-test or chi-square test.

### Prevalence of WRMSDs

Almost three quarters (73.24%) of the nurses reported having symptoms in at least one body region. Table 2 presents the12-months prevalence rates of WRMSDs by body region and shows that the neck was the most prevalent body region causing discomfort or pain (48.94%), followed by feet (47.20%), upper back (40.69%), shoulders (36.97%), and lower back (35.28%). Thighs and arms were listed as the body regions with the least common pain or discomfort with 19.36 percent and 6.63 percent, respectively. In addition, more than half of the nurses experienced multiple musculoskeletal pain and discomfort. Over half of the participants (54.82%) complained of pain in two body regions, 46.53 percent complained of pain in three body regions, while 35.43 percent reported pain in four or more body regions. Further examination of pain or discomfort in multiple body regions showed that more than half of the nurses experienced pain or discomfort in region one (neck, shoulders, and upper back) (59.74%) and region four (hips, knees, ankles, and feet) (52.45%), and only one out of four nurses claimed to have pain in region two (arms and wrists) (26.33%). Forty point one percent of nurses reported at least moderate pain (score of ≥ 3) in the neck, 34 percent reported moderate knee pain and only 1.6 percent of nurses complained of having pain in their arms for the past 12 months. Examination of factors associated with pain revealed that all demographic and work-related covariates, except for the number of years of employment, were significantly linked with pain at some of the body regions (p < 0.05). The older nurses suffered from discomfort at the lower limb (OR: 1.10, 95% CI: 0.94-1.24), whilst working an extra hour in the weekly working hours was associated with complaints reported in all body regions, except region one (shoulders, neck, and upper back). Nurses with higher BMI were significantly more likely to report pain in region two (upper limbs), compared to nurses with lower BMI (OR: 1.05, 95% CI: 1.00-1.11).

**Table 2:** Twelve-months prevalence of WRMSDs according to body region (N = 376)

**Body region**	**No. (%)**
Neck	184 (48.94)
Shoulders	139 (36.94)
Upper back	153 (40.69)
Arms	25 (6.63)
Wrists	99 (26.33)
Lower back	133 (35.28)
Thighs	73 (19.36)
Knees	96 (25.55)
Feet	178 (47.20)

### Psychosocial risk factors

The associations between job strain subscales were examined using Pearson’s correlation coefficients and were significantly associated. As expected, a modest correlation was observed between subscales of SS (supervisor support and co-worker support), r = 0.35. Both subscales also illustrated strong correlation with SS (r = 0.75-0.88). DL, PJD and SS showed positive correlation with each other (r = 0.27-0.34).Table 3 illustrates shows the mean scores of each psychosocial risk factor according to WRMSDs and shows that only one psychosocial risk factor, that of PJD, indicating a significant difference between individuals with and without WRMSDs (p < 0.05). In addition, the subscales were statistically associated with the occurrence of WRMSDs (p < 0.05). Based on the quadrant approach (median split), nurses with scores that exceeded the median value for PJD (>18.0) and below median for DL (<64.0), were categorized as being exposed to high strain job. One of three nurses (33.71%) were categorized as active workers, whereas 17.21%, 23.64%, and 25.44% were assigned as high, low and passive working groups, respectively. Further, nurses scoring below the median value for SS (<24.0), were identified as experiencing an iso strain. None of the job strain and iso strain group (median split) appeared to be significantly associated with WRMSDs. Next, based on quartile split (low, medium, high) [27], the percentage of nurses exposed to high strain was reduced to 7 percent whereas 3.5 percent experienced iso-strain and is statistically associated with the symptoms of WRMSDs, (p < 0.05). Despite differences in the calculation methods, the percentage appeared to be the same for iso strain (3.5 percent). Both job strain and iso-strain ratio displayed significant differences with respect to the WRMSDs pain (p < 0.05).

**Table 3:** Psychosocial risk factors by WRMSDs among nurses

**Psychosocial risk factors**	**WRMSDs [Mean (SD)]**			
	**Total (n = 376)**	**Yes (n = 275)**	**No (n = 101)**	**P-value**^**+**^
Decision latitude^a^	64.64 (6.09)	64.63 (6.28)	64.70 (5.55)	0.97
Psychological job demand^a^	18.34 (3.66)	18.09 (3.59)	19.00 (3.80)	0.03*
Social support^a^	23.88 (2.24)	23.85 (2.37)	24.93 (1.86)	0.44
Job insecurity^a^	6.33 (0.77)	6.35 (0.80)	6.30 (0.67)	0.59
	% (n)			
Job strain median split^b^	17.3 (65)	15.6 (43)	21.8 (22)	0.16
Job strain based on quartiles^b^	7.0 (28)	5.0 (15)	12.9 (13)	0.02*
Iso strain median split^b^	3.5 (13)	2.9 (8)	5.0 (5)	0.34
Iso strain based on quartiles^b^	3.5 (13)	2.0 (11)	4.0 (2)	0.33

*p < 0.05 indicate statistically significance differences.

^+^calculated by independent sample t test and Chi-square test.

^a^Continuous variables.

^b^Categorical variables.

Lastly, a multiple logistic regression analysis was performed to examine the associations between psychosocial risk factors and socio-demographic and occupational factors and the incidence of WRMSDs in the various body regions. Overall, the analysis demonstrated statistically significant associations between the various psychosocial risk factors and WRMSDs in different body regions (Table 4).

**Table 4:** Job Strain Model psychosocial risk factors of WRMSDs according to body regions in public hospital nurses

**Body region**	**Job Strain Model risk factors**^**a**^**[OR (95% Cl)]**				
	**Psychological job demand**	**Decision latitude**	**Co-worker support**	**Supervisor support**	**Social support**
Region 1^b^					
*Crude*	1.30 (0.86-1.96)	0.91 (0.60-1.38)	2.14 (1.01-4.53)*	1.76 (1.06-2.92)*	1.69 (1.05-2.74)*
*Adjusted*^f^	0.98 (0.91-1.06)	1.00 (0.91-1.08)	1.01 (0.86-1.19)	1.00 (0.93-1.08)	1.02 (0.93-1.11)
Region 2^c^					
*Crude*	1.00 (0.58-1.46)	1.19 (0.75-1.91)	1.28 (0.62-2.63)	1.28 (0.62-2.63)	1.44 (0.87-2.38)
*Adjusted*^f^	1.11 (1.02-1.21)*	1.12 (1.02-1.22)*	1.19 (1.01-1.41)*	1.07 (0.99-1.15)	1.10 (1.01-1.21)*
Region 3^d^					
*Crude*	1.52 (0.99-2.32)*	1.03 (0.67-1.58)	1.47 (0.75-2.88)	1.47 (0.75-2.88)	1.36 (0.85-2.17)
*Adjusted*^f^	1.02 (0.94-1.10)	1.06 (0.97-1.15)	1.03 (0.55-1.95)*	1.09 (1.01-1.18)*	1.06 (0.97-1.16)
Region 4^e^					
*Crude*	1.14 (0.76-1.71)	1.52 (1.01-2.29)*	1.11 (0.57-2.15)	1.11 (0.57-2.15)	1.17 (0.74-1.85)
*Adjusted*^f^	1.00 (0.92-1.08)	1.03 (0.95-1.12)	0.95 (0.83-1.09)	1.06 (0.99-1.14)	1.05 (0.96-1.15)
Any region					
*Crude*	1.42 (0.90-2.25)	1.00 (0.63-1.58)	1.77 (0.75-4.15)	1.58 (0.88-2.81)	1.45 (0.84-2.48)
*Adjusted*^f^	0.99 (0.91-1.08)	1.03 (0.93-1.13)	0.99 (0.83-1.18)	1.05 (0.95-1.15)	1.05 (0.95-1.17)

Note: ^a^Job Strain Model risk factors for WRMSDs using multiple logistic regression and expressed as Odds Ratio (OR) with 95% Confidence Interval (95% CI).

*p < 0.05 indicate statistically significance differences.

^b^Region 1 (neck, shoulders, and upper back), ^c^Region 2 (arms, wrists), ^d^Region 3 (lower back), ^e^Region 4 (hips, knees, ankles, and feet) ^f^Adjusted for age, BMI, weekly working hours, and years of employment.

Initially, the crude logistic regression demonstrated that poor SS was significantly associated with higher risk of pain (OR: 1.69, 95% Cl: 1.05-2.74) (p < 0.05) at region one (upper limb), similarly observed for insufficient peers and supervisors supports (OR: 1.76-2.14) (p < 0.05). The high demanding jobs was observed to significantly increased the pain risk in the region three (low back) (OR: 1.52, 95% Cl: 0.99-2.32) (p < 0.05). Also, the decision latitude yielded statistically significant risk for region four (lower extremities) (OR: 1.52, 95% CI: 1.01-2.29) (p < 0.05). Following, the adjusted odd ratios (95% Cl) of all the subscales showed statistically significant risk (OR: 1.03-1.19) (p < 0.05), with pain in region two (upper limb), except for those with low supervisor support. Whereas, both subscales of social support were observed to be significant risk factors amplified the pain in region three (low back) (OR: 1.03-1.09) (p < 0.05).However, none of the subscales showed significant risk associated with the prevalence of at least one body part, although yielded odds ratio more than one.

## Discussion

This study aimed to document the prevalence of self-perceived WRMSDs among nursing personnel working at public hospitals in Malaysia, and to determine the association between psychosocial risk factors and WRMSDs.

The findings indicated that almost three out of four nursing staff (73.2%), experienced pain or discomfort in at least one of any of body region for the past one year. The prevalence of WRMSDs in the current study was observed to be slightly higher than in Chinese nurses [8]. However, as compared to nursing personnel in other Asia countries, the prevalence was found to be much lower, (78.0%-94.6%) [5]–[7],[24]. Next, the prevalence of WRMSDs pain was examined by body region, which were categorised into four. The findings showed that region one (neck, shoulders, and upper back) was the most commonly reported region for pain (59.70%), followed by region three (lower back) (52.00%) while region two (upper limbs) was reported to have less pain (26.30%). Almost 50 percent of nurses suffered from neck pain, followed by discomfort at the knees (47.20%), upper back (40.69%), shoulders (36.97%), and lower back (35.28%), whereas only 6.63 percent experienced arm pain in the past one year. In general, the prevalence pattern for WRMSDs was similar to previous findings [5],[7],[8],[11],[12]. Ranking of pain according to body region in the present study was also similar to those reported in previous studies [1],[4],[5], except for pain involving the knees and lower back. In comparison, the prevalence of neck pain in the present study was much lower than those reported in previous studies among nurses in Japan (54.70%) [5] and Sweden (53.00%) [12]. Nevertheless, the prevalence appeared to be higher than reported among nurses in other counterparts (45.80%-46.30%) [7],[11] and in a local study (45.40%) [30]. Meanwhile, knee pain affected more than one third (47.20%) of the nursing population, higher than those reported in Iran (39.30%) [7] and China (34.40%) [8]. The upper back, the third most frequently reported WRMSDs (40.70%), was slightly higher than those reported in Japan [5], Sweden [12], and China [8]; nonetheless, the prevalence was lower than those reported in Iran (43.50%) [7]. The present study also reported a low prevalence (34.90%) for low back pain in comparison to higher levels (44.10%-80.00%) in other studies [5],[7],[12],[24],[31]. The discrepancy in the prevalence pattern was not unexpected and may be related to organizational and cultural diversity, as well as the individual’s perception of the pain [32].

Pain frequency, based on a five-point scale, ranging from “0” (no pain/never) to “4” (worst pain ever), was also examined in this study. One out of every three nurses reported at least moderate pain in the neck (40.0%) and feet (33.9%). There was limited previous literature on pain severity to make any meaningful comparison, which possibly relates to inconsistencies in the definition of the symptoms [7],[23],[27].

Overall, none of the personal factors (age, marital status, and BMI) or occupational factors were significantly associated with pain in any of the body region, similar to those reported in previous studies [33]. However, logistic regression test suggested an association between the factors including age, BMI and year of employment with the pain or discomfort in the body regions. Age was found to be significantly associated with the occurrence of pain in the region four (lower limbs) in agreement with earlier studies [22],[34]. The current study also showed a significant association between BMI and pain or discomfort in region two (upper limbs) (OR: 1.05, 95% CI: 1.00-1.11), corroborating previous findings, that person with high BMI is at greater risk of WRMSDs and possibly suffer multiple site pain [11],[22],[35]. In addition, nurses with longer work service were expected to experience greater risk of WRMSDs excepting region one (neck, shoulders, and upper back), which was similar to those reported elsewhere [36] but differs from nurses in Japan [5].

Similar to previous studies [7],[22],[27], the Malay validated Job Content Questionnaire (M-JCQ) [15],[26] was used to identify the psychosocial risk factors among nursing personnel in the current study. Two subscales; supervisor with co-worker support showed positive moderate correlation (0.4), in agreement with other studies [37]. PJD showed positive correlation with DL and SS, respectively, as showing previous studies [37]. On the other hand, an acceptable correlation was also observed between SS and DL as confirmed in other studies [37],[38]. The Cronbach’s alpha coefficient for SS was slightly higher (0.80) than those from a previous study among Asian women workers’ healthcare [38] (Cronbach’s α = 0.7). In addition, both subscales of DL (skill discretion and decision authority) scored an acceptable moderate values of Cronbach’s α = 0.70 and 0.60, similar to those reported in the Chinese study on healthcare among female staff (Cronbach’s α = 0.60) [38]. However, PJD displayed the lowest value of 0.50, in consonant with earlier studies [26],[37]. This scenario may possibly reflected that the items categorized under PJD subscales failed to portray the respective elements experienced among Asian workers, more accurately, Malaysian nurses, and improvements to the scale, should be considered in future research. Indeed, the confirmatory factor analyses supported the finding that the items for each subscales were loaded as expected [15],[26].

Meanwhile, the findings suggested that different psychosocial risk factors were significantly associated with pain at different body regions, as confirmed in earlier studies [8],[16],[17],[22]. Psychological job demand independently predicted significantly increased odds of musculoskeletal pain at region three (low back) (OR: 1.52, 95% CI: 0.99-2.32). Also, the adjusted odds ratio reached significant evidence of increasing the risk of WRMSDs at region two (upper limbs) (OR: 1.11, 95% CI: 1.02-1.21). The results of the analysis, corroborated the earlier findings observed among female service staffs (OR: 1.57, 95% CI: 1.14-2.20) [18] and among workers in various occupations (OR: 1.50, 95% CI: 0.80-2.80) [39]. In contrary, Lagerstrom et al. [16] found that PJD has increased the risk of neck and shoulder pain (OR: 1.65-1.82).

Both subscales of social support (co-worker support and supervisor support) predicted significant musculoskeletal pain at all body regions for both adjusted and crude odds ratio, excepting region four (lower limbs) (OR: 1.03-2.14) in accordance to the previous studies in Greece [9], Sweden [16] and China [8]. Likewise, Sembajwe et al. [22] also agreed that poor rapport with supervisors (OR: 0.58, 95% CI: 0.43-0.78) and high psychological job demand (OR: 1.98, 95% CI: 1.55-2.53) are significantly elevated the risk of multi-site WRMSDs. In contrast, a systematic review by Bongers et al. [40] suggested no consistent association between upper limbs and social support. Also, earlier studies among nursing personnel found that neither job demands [9],[16] nor low social support [8],[9] contributed significantly to the back, shoulder, and neck pain, although the odd risk value was more than 1, respectively. The results of the present research also proved that there is a significant positive relationship between job control in daily tasks with the occurrence of pain or discomfort in the region four (lower limbs) (OR: 1.52, 95% CI: 1.01-2.29) and in region two (upper limbs) after adjustment for potential covariates (OR: 1.12, 95% CI: 1.02-1.22), in agreement with previous research (OR: 1.73, 95% CI: 1.13-2.67) [16]. Nonetheless, Bongers et al. [17],[40] and Lagerstrom et al. [16] have found contradicted evidences on the association between job control and musculoskeletal disorders. However, in the current research, the psychosocial risk factors was not identified a significant risk factor to predict WRMSDs in at least one individual body area for the past one year in agreement with those in Japan [5] despite other studies documenting acceptable associations [16],[21].

This study has a number of limitations. First, the data obtained from the cross sectional design should be interpreted with caution as it is a difficult task to determine causality, i.e., whether the presence of factors contributed to the risk of sustaining WRMSDs or that the presence of WRMSDs resulted in psychosocial outcomes. Moreover, as the data were collected using self-reported techniques, participant responses may be biased as a result of social desirability to provide sociably favoured answers than the real experience [41]. Cultural and language differences may have possibly influenced the individuals’ understanding and interpretation on the study items [32]. In addition, the study was restricted to female nurses which may possibly create bias for certain gender preferences parameters for instance, in the psychological domain, females are generally found to have lower decision latitude than men in most populations [37].

## Conclusion

This study demonstrated the associations between psychosocial risk factors and prevalence of WRMSDs among nurses working in public hospitals. All subscales were proven to have significant roles to increase the risk at different body regions. The information shall serve as reference to further embark a prospective study, in such to scrutinize the causal role of the psychosocial risk factors. The components of psychosocial risk factors (job control, job demand and social support) shall be considered together with the strenuous work task when performing a comprehensive workplace assessment, formulating and implementing policy, in order to create a sustainable healthy working environment.

## Competing interests

The authors declare that they have no competing interests.

## Authors’ contributions

NA involved in the design of the study, carried out the data collection, performed the statistical analysis and drafted the manuscript. RBN participated in the design of the study, data analysis and helped in drafting the manuscript; QKF contributed in the design of the study and facilitated the process of preparing the manuscript; RMN involved in the study design and data collection, JO participated in the study design and assisted in the drafted manuscript. All authors read and approved the final manuscript.
